# From Critical Point to Critical Point: The Two-States Model Describes Liquid Water Self-Diffusion from 623 to 126 K

**DOI:** 10.3390/molecules26195899

**Published:** 2021-09-29

**Authors:** Carmelo Corsaro, Enza Fazio

**Affiliations:** Department of Mathematical and Computational Sciences, Physics Science and Earth Science, University of Messina, Viale F. Stagno D’Alcontres 31, I-98166 Messina, Italy; enfazio@unime.it

**Keywords:** water anomalies, extended dynamics, water diffusivity, hydrogen bonds, thermodynamic properties

## Abstract

Liquid’s behaviour, when close to critical points, is of extreme importance both for fundamental research and industrial applications. A detailed knowledge of the structural–dynamical correlations in their proximity is still today a target to reach. Liquid water anomalies are ascribed to the presence of a second liquid–liquid critical point, which seems to be located in the very deep supercooled regime, even below 200 K and at pressure around 2 kbar. In this work, the thermal behaviour of the self-diffusion coefficient for liquid water is analyzed, in terms of a two-states model, for the first time in a very wide thermal region (126 K < T < 623 K), including those of the two critical points. Further, the corresponding configurational entropy and isobaric-specific heat have been evaluated within the same interval. The two liquid states correspond to high and low-density water local structures that play a primary role on water dynamical behavior over 500 K.

## 1. Introduction

Liquid water shows many peculiar chemical physical properties that make it a unique compound. The best known anomaly is, of course, the density maximum at the temperature T = 277 K and atmospheric pressure. This is essentially explained by the presence of two different local structures having different average densities and symmetry [1,2] whose population depends on temperature [3]. In fact, an increase in temperature provokes a structural collapse (higher density) but, on the other hand, favours the volume expansion (lower density). Indeed, the high directionality of the hydrogen bonds (HBs) shows a strong tendency to “symmetry selection” and leads to the formation of locally favoured structures, such as the tetrahedral one. However, their formation is opposed by the van der Waals interactions that simply favour a denser configuration [4,5]. Therefore, the relative concentration of low-density liquid (LDL) structures increases on cooling, as is the opposite for that of high-density liquid (HDL) structures and fragments. The first experimental observation of the existence of low-density structures was performed in 2007 by Mallamace et al., who carried out FTIR spectroscopy experiments on liquid water confined in small hydrophilic silica pores and supercooled down to about 190 K [6]. They were able to assign and follow the infrared band relative to the LDL in liquid water, lying at essentially the same frequency (≃3120 cm${}^{-1}$) of the specific band corresponding to the low-density amorphous phase of water [7].

The anomalous properties of water observed up to date are about 74 but are progressively updated. The website by M. Chaplin constitutes a reference point for those who are interested in studying and keeping updated about the different aspects of water properties [8]. The main water thermodynamic anomalies at atmospheric pressure are: the minimum value assumed by the isobaric specific heat at about 308 K, the minimum value of the isothermal compressibility at about 319 K, and the negative values of the thermal expansion coefficient below 277 K (where it is null) [9]. It is worth mentioning that the thermal expansion coefficient is proportional to entropy and volume fluctuations, which are positively correlated for simple liquids, meaning that both increase with temperature. For water, it holds only for T>277 K, while, below this temperature, entropy and volume fluctuations are negatively correlated: the coefficient of thermal expansion becomes negative so that a volume increase provokes an entropy decrease. In addition, entropy and volume fluctuations show a net increase when cooling liquid water below T* ≃ 320 K down to the supercooled regime [10,11,12]. This temperature, which is only few degrees above the body temperature of mammals, coincides with the onset of the protein thermal unfolding [13]. Above it, water properties resemble those of normal liquids and this means that HBs are not stable, being that their lifetime too short. Instead, below it, the HB formation is energetically favoured, so that the peculiar water properties, or anomalies, are driven just by the HBs dynamics. The lower the temperature, the more enhanced the water anomalies that, in terms of thermodynamical response functions, give rise to a divergence-like behaviour in the deep supercooled regime. However, it seems now accepted that these functions, on cooling, reach a maximum value at about T${}_{L}≃$ 225 K and atmospheric pressure and then decrease again with the temperature [14]. This occurrence is still somehow debated and stays at the base of the proposed theoretical scenarios trying to explain water anomalous behavior [15]. Further, innovative experiments and advanced simulation techniques have recently given a strong support to the liquid–liquid phase transition scenario, invoking the existence of a second critical point in water [15,16,17,18,19,20,21,22]. It is worth mentioning that other liquids with a tetrahedral structure, such as Si, SiO${}_{2}$, Ge, C, GeO${}_{2}$, and BeF${}_{2}$, show anomalies similar to those of water, although they do not form HBs [23]. Therefore, the tetrahedral symmetry mainly contributes to determining the thermodynamical anomalies of these compounds. Nevertheless, theoretical studies suggested that the water anomalous behaviour can be described as a hierarchy of factors of different nature [24]. Structural, dynamical, and thermodynamical effects can determine, depending on the temperature and density values, the water’s chemico-physical properties that, as it is well-known, influence those of the systems with which it interacts [25].

In this work, we focus on water’s dynamical behaviour in a very broad thermodynamical region going from the temperature of the known critical point (647 K) to that (and even below) of the hypothesized second critical point (≤200 K) [15,17]. In particular, for the first time, we analyze the experimental diffusion data of liquid water in a wide temperature interval (623–126 K) by using the two-states model, recently applied for successfully describing liquid water self-diffusion from 373 K down to the very deep supercooled regime (126 K) [26] and validated by using computational approaches [27]. Our effort is to extend the analysis to the high temperature critical region in view of potential technological applications, such as the optimization of the operating conditions of supercritical water reactors exploited in thermochemical conversion of biomass into biofuel [28,29]. Moreover, the knowledge of water’s supercritical condition is useful for managing catalysts, sorbents, or membranes for separation processes, such as desalination or nutrient retrieval during the mentioned biofuel production [30,31]. Note that investigating liquid water at these high temperatures can be possible by moving along the liquid branch of the coexisting curve, so pressure changes as well [32].

## 2. Results and Discussion

Translational diffusion is the most fundamental form of molecular transport, driven by the kinetic energy of the diffusing system. The value of the self-diffusion coefficient is strictly connected to the size of the diffusing object and the viscosity of the system (solution) in which the diffusion takes place. This connection is given by the well-known Stokes–Einstein relation (SER):

(1) $$D=\frac{k_{B}T}{\zeta }$$

In the friction term ζ, both the size of the diffusing object and the viscosity η of the “solution” are included. For a spherical molecule of hydrodynamic radius $R_{H}$, it equals $6\pi \eta R_{H}$; then, for a normal liquid, the SER can be written as:

(2) $$\frac{D\eta }{T}≃const.$$

It is worth mentioning that this relation breaks down at temperatures not far above the system’s glass transition temperature $T_{g}$ [33]. Instead, for water, the violation of SER seems to set on (at about 225 K, where D takes on the value of about 2.2 × 10${}^{-11}$ m${}^{2}$/s and η of about 2000 mPa s [34]) well above the glass transition temperature, predicted to be at ≈136 K [35]. This could be due to the peculiar role of HBs in enhancing the development of dynamical heterogeneities [36,37]. These are associated with the growth of large clusters in the LDL phase of water, causing the decoupling of transport properties once the Widom line is crossed. The Widom line is defined as the critical isochore departing from the hypothesized liquid–liquid critical Point (LLCP) that defines the locus of a liquid–liquid transition (LLT) between two local water structures with different densities [26,38,39]. The Widom line is, in reality, a bundle of lines that emanates from the LLCP and, here, we will refer to that corresponding to the specific heat [40]. The existence of the LLT has been investigated by both experimental and theoretical studies, arguing that its occurrence does not depend on the system’s dimensionality [41] or nature (it has also been observed for hydrated proteins and DNA [42,43]), whereas its corresponding temperature is somehow influenced by the solute concentration [44,45].

Several theories and theoretical approaches have been developed to explain the complex behaviour of liquids (including water) when cooled below their melting temperature [46]. The most accredited include the mode-coupling theory (MCT) [47,48], the Adam–Gibbs (AG) theory [49], and the potential energy landscape [50,51]. The MCT is the only purely dynamical approach, based on a microscopic theory of the dynamics of fluids, that treats the glass transition on mean-field bases. Instead, the Adam–Gibbs theory describes a connection between dynamics and thermodynamics. For the AG theory, the self-diffusion coefficient $D_{s}$ can be expressed in terms of the configurational entropy $S_{conf}$ as:

(3) $$D_{s}=D_{0}\exp\left(\frac{A}{TS_{conf}}\right)$$

where A=−31.6 kJ/mol and $D_{0}=1.07\times 10^{-7}$ m${}^{2}$/s are constant [52,53]. The AG theory is founded on arguments related to the concept of cooperatively rearranging regions (CRR), in which the relaxation processes are cooperatively in character. On cooling, the size of the CRR increases, causing the configurational entropy to decrease and the dynamics to slow down [54]. These CRR can be associated with the so-called dynamic heterogeneities, which are regions of the system characterized by different collective dynamics (with respect to the average) and ascribed to be the imputato for the decoupling between rotational and translational dynamics, expressed as the violation of the Stokes–Einstein relation [55].

**Figure 1:**
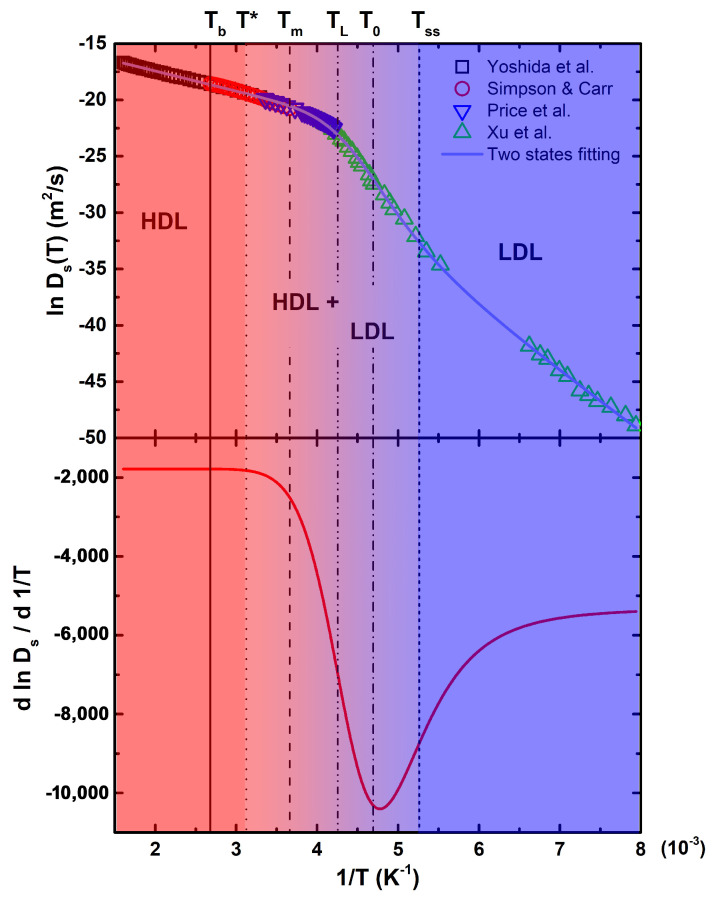
The natural logarithm of the self-diffusion coefficient for liquid water (**top**), fitted with the two-states model and the corresponding first derivative, with respect to the inverse temperature (**bottom**) in a wide thermal region 126 K < T < 623 K. Vertical lines refer to the relevant temperatures discussed in the text.

The temperature dependence of water translational diffusion is shown in Figure 1, reporting data from 623 down to 126 K and covering the largest temperature interval of every previously published diffusion data on liquid water [56]. Here, we have considered four sets of data, those by: (i) Yoshida et al. [32], from 623 to 303 K, who performed NMR measurements along the liquid branch of the coexistence curve; (ii) Simpson and Carr, from 373 to 273 K [57], and (iii) Price et al., from 298 to 238 K [58], who both used pulsed field gradient NMR; and (iv) Xu et al., from 262 to 126 K [59], who adopted a pulsed laser heating technique for the melting of ice. Vertical lines refer to the following relevant temperatures for water: $T_{b}=373$ K is the boiling temperature; $T^{*}≃320$ K corresponds to the onset of stable HBs [60]; $T_{m}=273$ K is the melting temperature; $T_{L}≃235$ K represents the locus of the liquid–liquid transition, identified by Xu et al. [59]; $T_{0}=213$ K, found by the two-states fitting, corresponds to the temperature at which the populations of HDL and LDL are equal; and $T_{ss}≃180$ K is the temperature of a strong-to-strong dynamical crossover [59].

The bottom panel of Figure 1 reports the thermal derivative of the two-states fitting of the natural logarithm of self-diffusion, allowing for a better visualization of the temperatures at which dynamical changes occur. Note that $T^{*}$ corresponds to the first deviation from the constant value at the highest temperatures, suggesting that HBs become relevant just below it. The temperature of the Widom line, departing from the hypotesized LLCP being the locus of the maximum correlation length, lies between $T_{L}$ and $T_{0}$. The solid line across all the experimental points is a fitting with a two liquids model recently used by Skinner et al. [26] and Piskulich et al. [61]. In this model, the temperature dependence of the self-diffusion coefficient is expressed as a weighted sum of two separate Arrhenius contributions belonging to HDL and LDL states of liquid water:

(4) $$\ln D_{s}\left(T\right)=s\left(T\right)\ln D_{L}\left(T\right)+\left[1-s\left(T\right)\right]\ln D_{H}\left(T\right)$$

in which the subscripts *H* and *L* stand for HDL and LDL, respectively, so that the corresponding self-diffusion coefficients can be written in an Arrhenius form:

(5) $$D_{L}\left(T\right)=D_{0,L}\exp\frac{-E_{a,L}}{k_{B}T}\;;\;D_{H}\left(T\right)=D_{0,H}\exp\frac{-E_{a,H}}{k_{B}T}$$

where both are defined in terms of the Arrhenius activation energy, $E_{a}$, and diffusion amplitude $D_{0}$. The weighting function s(T) takes into account switching between the two dynamical regimes:

(6) $$s\left(T\right)=\frac{1}{1+\exp\frac{a(T-T_{0})}{\Delta T}}$$

in which $T_{0}$ corresponds to the temperature at which the two contributions have the same weight (s(T)=0.5) and *a* is assumed to be equal to 4.394, such that ΔT defines the thermal interval for which s(T) goes from 0.1 to 0.9. This temperature interval, where HDL and LDL coexist, essentially lies within the range of validity of the MCT power-law behaviour, in which fluctuations are strongly coupled.

The fitting results, reported in Table 1, agree with those found by Skinner et al. [26] and Piskulich et al. [61], although they considered a smaller temperature interval. Therefore, our results not only confirm the effectiveness of this approach but also the extend its validity up to the water critical region. The next step will be a proper interpretation of the switching function, for example, in terms of hydrogen bonds strength and corresponding temperature dependence. We are developing a model that explicitly includes the thermal dependence of HBs strength and distance (network reorganization), also taking into account what reported in the references [62,63].

**Figure 2:**
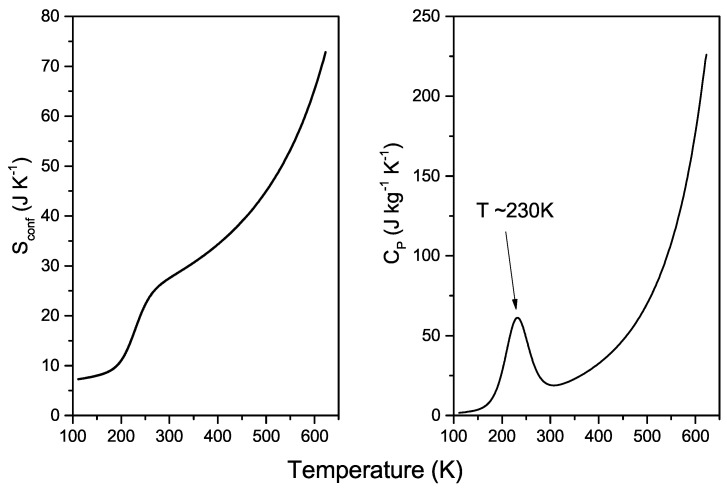
The configurational entropy contribution, obtained by means of the AG relation (Equation (3)), applied to the two-states fitting of water self-diffusion (**left panel**). The corresponding configuration-specific heat, evaluated by using Equation (7) (**right panel**).

Now, by using the AG relation (3), we first evaluate the configurational entropy (Figure 2 left panel) from the D${}_{s}$(T) fitting curve and then the corresponding configuration-specific heat (Figure 2 right panel) by using:

(7) $$c_{P,conf}=T\left(\frac{\partial S_{conf}}{\partial T}\right)$$

As is known, the entropy contribution is of fundamental importance in both chemical and biological processes involving water. The hydrophobic effect, molecular recognition, and ligand binding are only few examples of this [64,65]. The thermal behaviour of $S_{conf}$ increases monotonically with temperature, showing a flex point at about 230 K (Figure 2 left panel). This monotonic increase with temperature corresponds to the progressive loosening of local molecular order on heating. The occurrence of the inflection point in $S_{conf}$ coincides with the temperature of the population inversion between local high-density and low-density structures on cooling [6]. Subsequently, the evaluated configuration-specific heat displays a well-defined maximum at the same temperature, which is in agreement with the LLCP hypothesis [53,66] and goes toward a diverging-like behaviour along the liquid-vapour coexistence line. It is worth mentioning that for pressures higher than the critical one (P${}_{C}$ = 220.6 bar), a Widom line, where specific heat assumes its maximum value, departs from the high temperature critical point [67].

The thermodynamic implications regarding the existence of two main, different liquid states with different densities must take into consideration the hydrogen bond ability of water molecules. It is now demonstrated that HBs can also form in supercritical conditions, but no HB networks can exist at all [67,68,69]. The knowledge of dynamic and thermodynamic quantities, including $S_{conf}$ and $c_{P,conf}$ at temperatures as high as the critical one, is essential both for fundamental research and industrial applications. Supercritical water is a cheap, inorganic, and green solvent that can be successfully employed in many processes, such as biomass processing [70,71], chemical synthesis [72], carbon capture, and storage [73]. Its low dielectric constant and high solubility make it a suitable and homogeneous single-phase solvent for organic synthesis reactions [72]. Furthermore, its peculiar properties also have a main role in geological processes, such as the dissipation of subduction slabs and the melting point decrease of the neighboring rocks leading to the formation of magma and volcanism in subduction zones [74]. Finally recent applications consider the construction of an efficient nuclear plant by taking advantage of the thermophysical properties of water in the supercritical region [75,76]. The analysis of the behaviour of relevant thermodynamic quantities, including entropy and specific heat, is therefore a mandatory step for the optimization of the mechanisms involved in the considered processes. Therefore, as also evidenced by our study, the positive changes, shown on heating by configurational entropy (Figure 2 left panel), correspond to a progressively less ordered structure (with higher density) and greater hydrogen bond breaking [77].

## 3. Conclusions

The coexistence of local structures in liquid water with different densities determining its dynamical and thermodynamical properties is nowadays well demonstrated. Temperature and pressure values shift the relative populations so defining a complex phase diagram. The dynamic scenario can be interpreted in terms of two Arrhenius behaviours with different activation energies observable in different temperature intervals. For temperatures higher than T*∼ 320 K, only high-density, local structures are present up to the well-known critical point region. Whereas, for temperatures lower than *T*${}_{0}∼$ 213 K, only low-density local structures exist down to the liquid–liquid critical point region. The understanding of water behaviour within critical regions is pivotal for innovative applications involving chemical, biological, and geological processes, thanks to its peculiar physical and chemical properties. Future perspectives include the physical interpretation of the weighting function and its correlation to arguments, such as the tetrahedrality. In such a way, a more confident comprehension of hydrogen bonds dynamics at atomic and molecular level can be achieved, with the aim to also include quantum phenomena, such as the ultrafast hydrogen bond strengthening observed only in liquid water [78].

## Figures and Tables

**Table 1:** Fitting results of the two-states model applied to the water self-diffusion data, reported in Figure 1.

D${}_{0,H}\;(10^{-7}$ m${}^{2}$ s${}^{-1})$		D${}_{0,L}\;(10^{-4}$ m${}^{2}$ s${}^{-1})$		E${}_{a,H}\;$ (kcal mol${}^{-1}$)		E${}_{a,L}$ (kcal mol${}^{-1}$)	
**Value**	**Stnd Err**	**Value**	**Stnd Err**	**Value**	**Stnd Err**	**Value**	**Stnd Err**
9.50	0.71	9.48	0.89	3.56	0.05	10.64	0.19
**T${}_{0}\;\left(K\right)$**		** ΔT(K) **		**Statistics**			
**Value**	**Error**	**Value**	**Error**	**Red Chi-Sqr**	**Adj. R-Sqr**		
212.8	1.0	71.3	1.9	0.016	0.999		

## Data Availability

The data presented in this study are available on request from the corresponding author.

## Abbreviations

The following abbreviations are used in this manuscript:

HB	Hydrogen Bond
LDL	Low-Density Liquid
HDL	High-Density Liquid
LLCP	Liquid–Liquid Critical Point
LLT	Liquid–Liquid Transition
MCT	Mode-Coupling Theory
AG	Adam–Gibbs
CRR	Cooperatively Rearranging Regions
